# Paeonol Attenuates UVB-Induced Premature Senescence in Human Dermal Fibroblasts to Promote Skin Longevity

**DOI:** 10.3390/ijms27209352

**Published:** 2026-10-21

**Authors:** Kyuri Lee, Jeung hi Han, Hairu Zhao, Young Eun Kim, Yang Gyu Choi, Hosam Ki, Jaehong Kim, Young Jin Choi, Hye Yoon Park, Areum Jang, Min Hyuk Park, Hang Eui Cho, Jae Sung Hwang

**Affiliations:** 1 Department of Genetics & Biotechnology, Graduate School of biotechnology, College of Life Sciences, Kyung Hee University, Yongin 17104, Republic of Korea; kyuri.lee@khu.ac.kr (K.L.); zhao@khu.ac.kr (H.Z.); 2 Research and Innovation Center, LABIO Co., Ltd., Seoul 08501, Republic of Korea; msri05@labio.kr (J.h.H.); msri14@labio.kr (Y.E.K.); msri22@labio.kr (Y.G.C.); msri07@labio.kr (H.K.); msri06@labio.kr (J.K.); 3 Cosmecca Korea Co., Ltd., Seongnam 13488, Republic of Korea; cyj0120@cosmecca.com (Y.J.C.); rejoice077@cosmecca.com (H.Y.P.); areumnamoo@cosmecca.com (A.J.); alsgur0728@cosmecca.com (M.H.P.)

**Keywords:** paeonol, UVB-SIPS, cellular senescence, skin longevity, HDFn, premature senescence

## Abstract

Skin photoaging is largely driven by ultraviolet (UV) irradiation, which contributes to chronic inflammation and extracellular matrix (ECM) degradation. This study investigated the protective effects of paeonol against UVB-induced cellular senescence in human dermal fibroblasts (HDFn). Repeated UVB irradiation was used to induce premature senescence. Paeonol treatment reduced the senescence-associated β-galactosidase (SA-β-gal) positive cells and intracellular reactive oxygen species (ROS) accumulation. Paeonol also reduced the expression of senescence-associated cell cycle regulators, including p21 and p16 at the mRNA level and the protein level. In addition, paeonol suppressed matrix metalloproteinase-1 (MMP-1) production but did not significantly restore type I procollagen secretion. Paeonol also attenuated the expression and secretion of the senescence-associated secretory phenotype (SASP) factors inter-leukin-6 (IL-6) and interleukin-8 (IL-8). These effects were accompanied by reduced phosphorylation of JNK and c-Jun. Furthermore, activation of JNK signaling with anisomycin partially reversed the paeonol-mediated suppression of MMP-1 mRNA expression, supporting the involvement of JNK signaling in the regulation of MMP-1. Collectively, these findings suggest that paeonol attenuates multiple features of UVB-induced cellular senescence in HDFn cells, with JNK signaling potentially contributing to its inhibitory effect on MMP-1 expression. Further studies are needed to clarify the mechanisms underlying these effects and establish their physiological relevance in UVA-based and in vivo models.

## 1. Introduction

Skin aging is a complex process caused by the cumulative effects of intrinsic and extrinsic stress. Intrinsically aged skin displays thin and mildly wrinkled features, while photoaged skin is characterized by a thickened epidermis, deep wrinkles, irregular pigmentation, and disorganization of dermal connective tissue [1]. Skin research has recently moved toward the idea of “skinspan” or “skin longevity” to overcome these structural alterations and prevent aging. This idea aims to preserve the skin’s structural integrity and healthy function throughout life [2,3]. Dermal fibroblasts play a crucial role in maintaining skin homeostasis by regulating extracellular matrix (ECM) synthesis and remodeling. However, the accumulation of senescent fibroblasts can disrupt tissue homeostasis through altered ECM metabolism and the secretion of senescence-associated secretory phenotype (SASP) factors, thereby contributing to skin aging [2,4].

Cellular senescence is recognized as one of the main hallmarks of aging, contributing to tissue dysfunction and age-related diseases [5,6]. Senescent cells enter a stable cell-cycle arrest and, although they remain metabolically active, they permanently lose their ability to divide [7,8]. This arrest is enforced mainly through two pathways, the p53/p21^WAF1^ axis and the p16^INK4a^/pRb axis, which inhibit cyclin-dependent kinases (CDKs) and thereby maintain the retinoblastoma protein (pRb) in a hypo-phosphorylated, active state [6,7,9,10,11]. A further defining feature of senescent cells is their resistance to apoptosis. This allows them to persist as dysfunctional cells within the tissue instead of being eliminated [12,13]. Since cleavage of poly (ADP-ribose) polymerase (PARP) by activated caspases is a known biochemical marker of apoptosis [14.^15^], the apoptotic versus senescent fate of stressed cells can be discriminated on this basis.

Ultraviolet (UV) radiation is one of the extrinsic stressors driving skin aging, also known as photoaging [2,16,17]. Photodamage to skin can lead to wrinkles, hypo- or hyper-pigmentation and higher chance of developing skin cancer. UV radiation consists of UVA (315–400 nm), UVB (280–315 nm), and UVC (100–280 nm). However, only UVA and UVB reach the skin. These wavelengths differ in their tissue penetration and mechanism of action. UVA accounts for approximately 95% of the solar UV that reaches the Earth’s surface and penetrates deeply into the reticular dermis. It stimulates reactive oxygen species (ROS) generation [18,19,20]. Through oxidative stress, UVA activates MAPK/AP-1 signaling and induces MMP-1 expression in dermal fibroblasts, leading to collagen degradation [21,22]. Chronic UVA exposure, together with UVB, can damage the dermal ECM and contribute to wrinkle formation [23,24,25].

Although UVB accounts for a smaller proportion of UV radiation that reaches the Earth’s surface, it is more effective in inducing cutaneous biological effects [19,20]. UVB acts primarily through the direct absorption by cellular chromophores such as DNA, and to a lesser extent through ROS generation [19]. UVB is absorbed mostly by the epidermis but a fraction reaches the upper papillary dermis [18,19,20,23]. Beyond its damaging effects, UV radiation regulates the cutaneous neuro-immuno-endocrine system, inducing local production of hormones, neuropeptides, and cytokines that influence systemic homeostasis [19,20,26].

Among the diverse responses to UVB, the present study focused on stress-induced premature senescence (SIPS) of dermal fibroblasts, which is induced by repeated exposure to subcytotoxic UVB [1,27,28,29]. This repeated UVB-SIPS model is well-established for studying multiple senescence hallmarks [29,30,31] and remains in use [23]. Accordingly, this study investigated the protective effects of paeonol in this UVB-SIPS model, rather than reproducing the full spectrum of solar radiation or all mechanisms underlying in vivo photoaging.

Senescent cells secrete a senescence-associated secretory phenotype (SASP), characterized by the release of chemokines, matrix-degrading enzymes, and pro-inflammatory cytokines such as IL-6 and IL-8 [10,32]. These SASP factors can reinforce the senescent phenotype through autocrine signaling and also induce senescence in nearby cells through paracrine signaling [7,10,32]. Matrix metalloproteinases (MMPs) are the principal collagenases that degrade collagens. In senescent cells, MMPs become upregulated [16,33]. Mechanistically, UVB-induced ROS activate the mitogen-activated protein kinase (MAPK) cascade, including c-Jun N-terminal kinase (JNK), which subsequently activates the activator protein-1 (AP-1) transcription complex. This AP-1 activation increases the expression of MMP-1, MMP-3, and MMP-9 [21,34,35]. Ultimately, senescence and SASP-associated changes contribute to continuous extracellular matrix (ECM) degradation and chronic inflammation. This leads to structural deterioration of the skin.

Bioactive compounds are considered potential candidates for cosmetics and dermatology applications due to their antioxidant and anti-inflammatory activities [4,36,37,38]. To identify compounds with potential protective effects against UVB-SIPS, we conducted a preliminary screening using various natural compounds and selected paeonol (2′-hydroxy-4′-methoxyacetophenone) for further investigation. Paeonol is a bioactive compound isolated from the root bark of Paeonia suffruticosa (Cortex Moutan), a plant traditionally used in East Asian medicine [39]. Previous studies have reported antioxidant, anti-inflammatory, and anti-aging effects of paeonol in various cell types [39,40]. Notably, Cortex Moutan extracts have been shown to suppress MMP-1 production in UVB-irradiated human dermal fibroblasts [41]. In addition, paeonol alone has been reported to attenuate H_2_O_2_-induced senescence in human fetal lung fibroblasts, reducing SA-β-gal positive cells and intracellular ROS. Furthermore, paeonol suppressed UVB-induced MAPK/AP-1 activation and MMP-1 production in human keratinocytes [42,43].

Despite these previous findings, the effects of paeonol on UVB-induced senescence in human dermal fibroblasts remain insufficiently characterized. Therefore, in this study, we aimed to evaluate the effects of paeonol on the UVB-SIPS model and the SASP in human dermal fibroblasts.

## 2. Results

### 2.1. Confirmation of the UVB-Induced Senescence Model and Dose Selection

**Figure 1:**
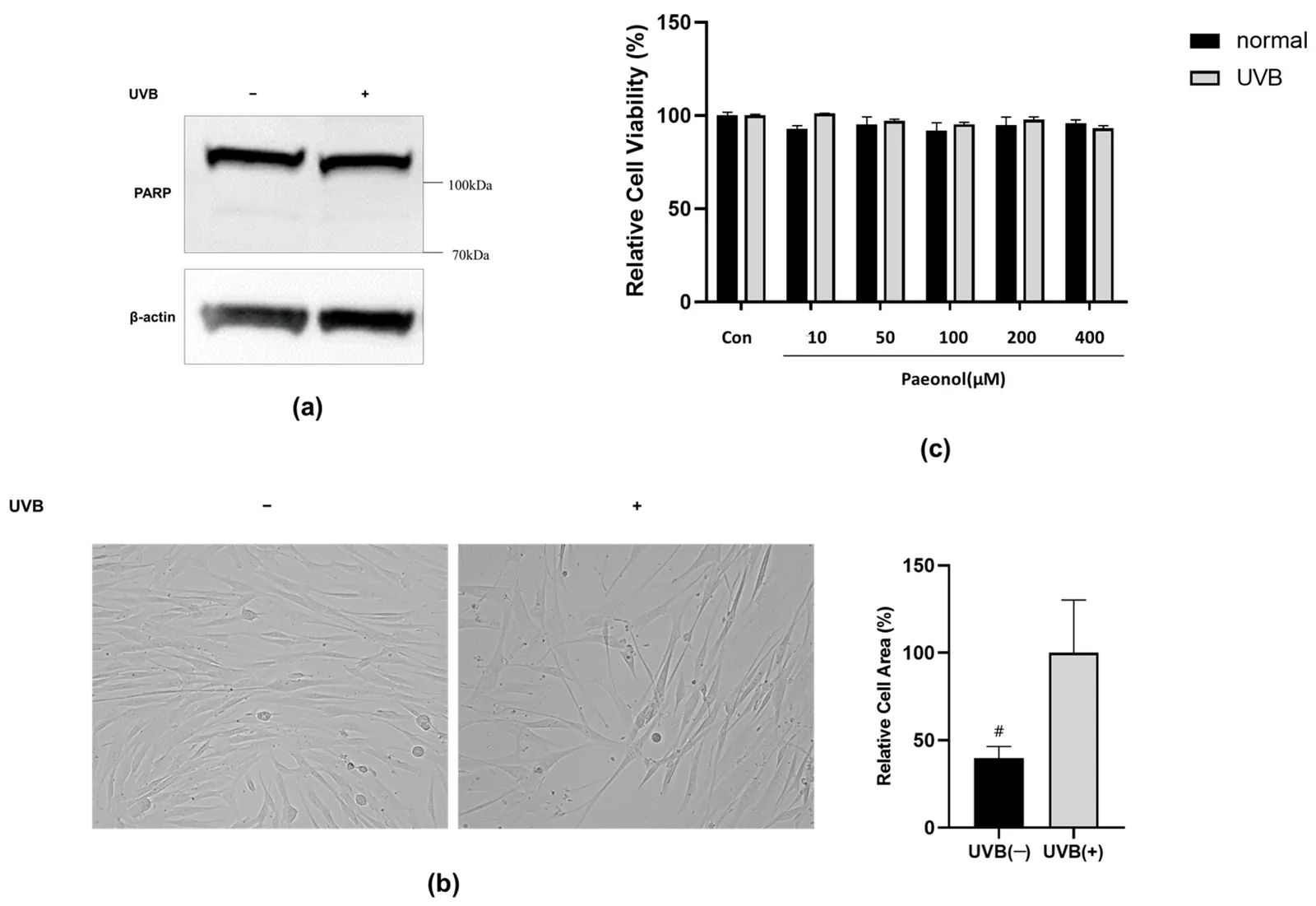
Confirmation of UVB-induced senescence without apoptosis and evaluation of paeonol cytotoxicity in HDFn cells. Senescence was induced by repeated UVB irradiation (250 mJ/cm^2^, once daily for 4 consecutive days). (**a**) Western blot analysis of PARP in normal (UVB −) and senescent (UVB +) HDFn cells, with β-actin as the loading control. Full-length PARP was detected in both groups, whereas the cleaved PARP fragment was not detected, indicating that repeated UVB irradiation induced senescence rather than apoptotic cell death. (**b**) Phase-contrast images of normal (UVB −) and senescent (UVB +) HDFn cells, showing the shift from a spindle-shaped to an enlarged, flattened senescent phenotype, with morphometric quantification of cell area measured using ImageJ. For each of three independent experiments, the mean cell area was calculated from clearly delineated cells within a representative field, and the per-experiment means were compared between groups. Original magnification, ×10. (**c**) Effect of paeonol on the viability of normal and UVB-irradiated HDFn cells. The cells were treated with the indicated concentrations of paeonol (10–400 μM) for 24 h, and cell viability was measured using the EZ-Cytox assay. Data are presented as the mean ± SD (n = 3). # *p* < 0.05 vs. the normal control group.

To confirm that the UVB-SIPS model used in this study induces cellular senescence without triggering apoptosis, the expression levels of full-length and cleaved PARP were examined by Western blotting in HDFn cells. No noticeable cleaved PARP band was detected, whereas full-length PARP was present at comparable levels (Figure 1a). This indicates that the current UVB irradiation method successfully induces stress-induced premature senescence (SIPS) rather than apoptotic cell death.

UVB-irradiated HDFn cells displayed the characteristic morphological shift from a spindle-shaped to an enlarged, flattened appearance compared with normal cells (Figure 1b). Quantitative morphometric analysis confirmed this shift. The mean cell area of UVB-irradiated fibroblasts was significantly greater than that of normal cells (Figure 1b).

To determine the safe working concentration range of paeonol, a cell viability assay was conducted. HDFn cells were treated with various concentrations of paeonol under both normal and UVB-induced senescent conditions (Figure 1c). Paeonol did not significantly affect cell viability at any of the tested concentrations under either condition. Based on these results and our preliminary experiments, 10 µM showed no significant protective effect compared with the UVB control group, whereas 200 µM tended to increase cellular stress. Therefore, 50 µM and 100 µM were selected as the optimal concentrations for subsequent experiments.

### 2.2. Paeonol Attenuates UVB-Induced MMP-1 Production and Intracellular ROS Accumulation but Does Not Restore Type I Procollagen Secretion

**Figure 2:**
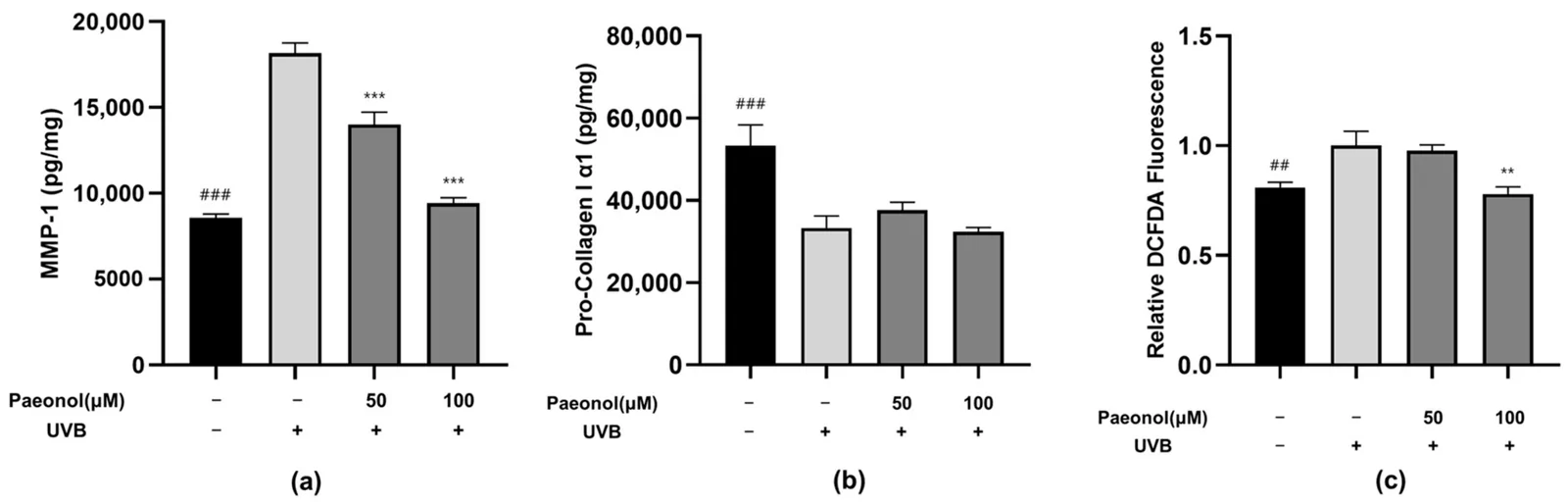
Paeonol attenuates UVB-induced MMP-1 secretion and intracellular ROS accumulation but does not restore Type I procollagen secretion in HDFn cells. Senescence was induced by repeated UVB irradiation (250 mJ/cm^2^, once daily for 4 consecutive days). (**a**) Secreted MMP-1 levels. Culture supernatants were collected 24 h after replating, and MMP-1 was quantified by ELISA. (**b**) Secreted Type I procollagen (procollagen Iα1) levels quantified by ELISA; senescent cells showed reduced procollagen secretion relative to normal cells, which was not restored by paeonol. (**c**) Intracellular ROS levels. Cells were stained with DCFDA, and the relative fluorescence intensity was measured. Cells were divided into the normal control (no UVB, no treatment), UVB control (UVB only), and paeonol treatment groups (50 and 100 µM). Data are presented as the mean ± SD (n = 3). ## *p* < 0.01, ### *p* < 0.001 vs. the normal control group; ** *p* < 0.01, *** *p* < 0.001 vs. the UVB control group.

UVB irradiation significantly increased MMP-1 secretion compared with the normal control group. However, paeonol treatment at 50 µM and 100 µM significantly decreased UVB-induced MMP-1 production in a dose-dependent manner (Figure 2a).

Because dermal extracellular matrix (ECM) loss during senescence results from both increased MMP-mediated collagen degradation and reduced collagen synthesis, we next examined Type I procollagen secretion. UVB-induced senescent HDFn cells secreted significantly lower levels of Type I procollagen than normal cells, reflecting the impaired collagen-synthetic capacity of senescent fibroblasts. In contrast to its robust suppression of MMP-1, however, paeonol did not significantly restore procollagen secretion (Figure 2b). These results indicate that paeonol preserves the dermal ECM primarily by limiting collagen degradation rather than by promoting collagen synthesis.

We further assessed intracellular ROS, the principal upstream trigger of UVB-induced senescence. The UVB-irradiated cells exhibited increased intracellular ROS levels compared with the normal control group, whereas paeonol treatment at 100 µM attenuated UVB-induced ROS accumulation (Figure 2c).

### 2.3. Paeonol Suppresses UVB-Induced Senescence Markers and Senescent Cell Burden

**Figure 3:**
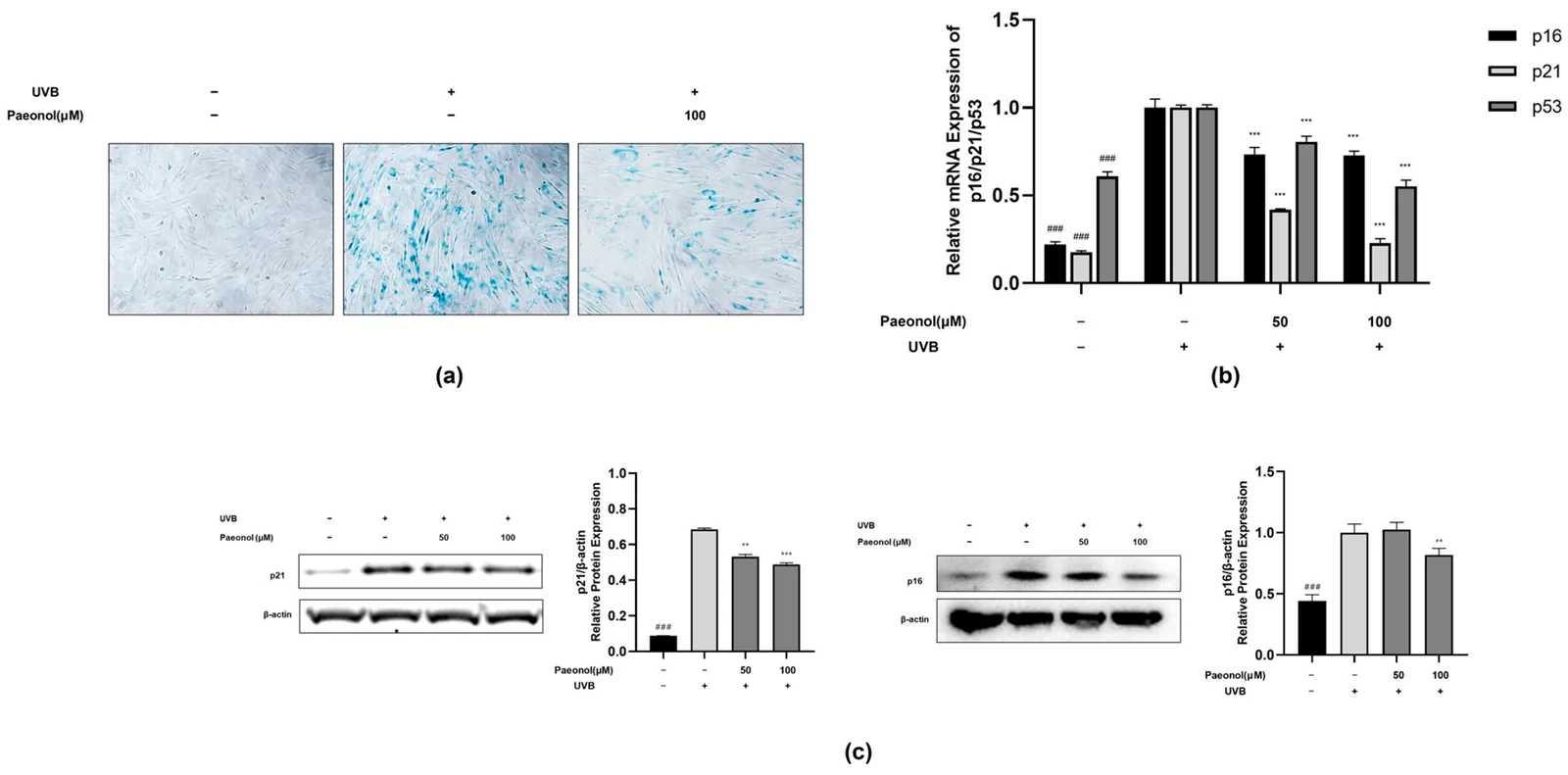
Paeonol suppresses UVB-induced senescent cell burden and senescence markers in HDFn cells. Senescence was induced by repeated UVB irradiation (250 mJ/cm^2^, once daily for 4 consecutive days). (**a**) Representative images and quantification of SA-β-gal staining; UVB increased the proportion of SA-β-gal-positive cells, which was reduced by paeonol. (**b**) Relative mRNA expression levels of p21, p16, and p53 measured by RT-qPCR; β-actin was used as the internal reference gene. (**c**) Protein expression levels of p21 and p16 determined by Western blot analysis; band intensities were quantified and normalized to β-actin. For (**a**), paeonol was applied at 100 µM; for (**b**,**c**), the cells were divided into the normal control, UVB control, and paeonol treatment groups (50 and 100 µM). Data are presented as the mean ± SD (n = 3). ### *p* < 0.001 vs. the normal control group; ** *p* < 0.01, *** *p* < 0.001 vs. the UVB control group.

To evaluate the effect of paeonol on cellular senescence at the phenotypic level, SA-β-gal staining was performed using 100 µM paeonol, the most effective concentration in the preceding experiments. UVB irradiation markedly increased the proportion of SA-β-gal-positive cells compared with the normal control group, whereas paeonol treatment significantly reduced the senescent cell fraction (Figure 3a).

To define the molecular basis of this effect, we measured the mRNA and protein expression of senescence markers by RT-qPCR and Western blot analysis. UVB irradiation increased the mRNA expression of p21, p16, and p53 compared with the normal control group, whereas paeonol treatment reduced the mRNA levels of all three markers in a dose-dependent manner (Figure 3b). Consistent with the transcript results, Western blot analysis showed that UVB increased the protein levels of p21 and p16—key downstream regulators of cell cycle arrest—relative to the normal control group, and paeonol treatment decreased both (Figure 3c). Together, these results indicate that paeonol suppresses UVB-induced senescence at the cellular, transcriptional, and translational levels.

### 2.4. Paeonol Modulates the Senescence-Associated Secretory Phenotype (SASP)

**Figure 4:**
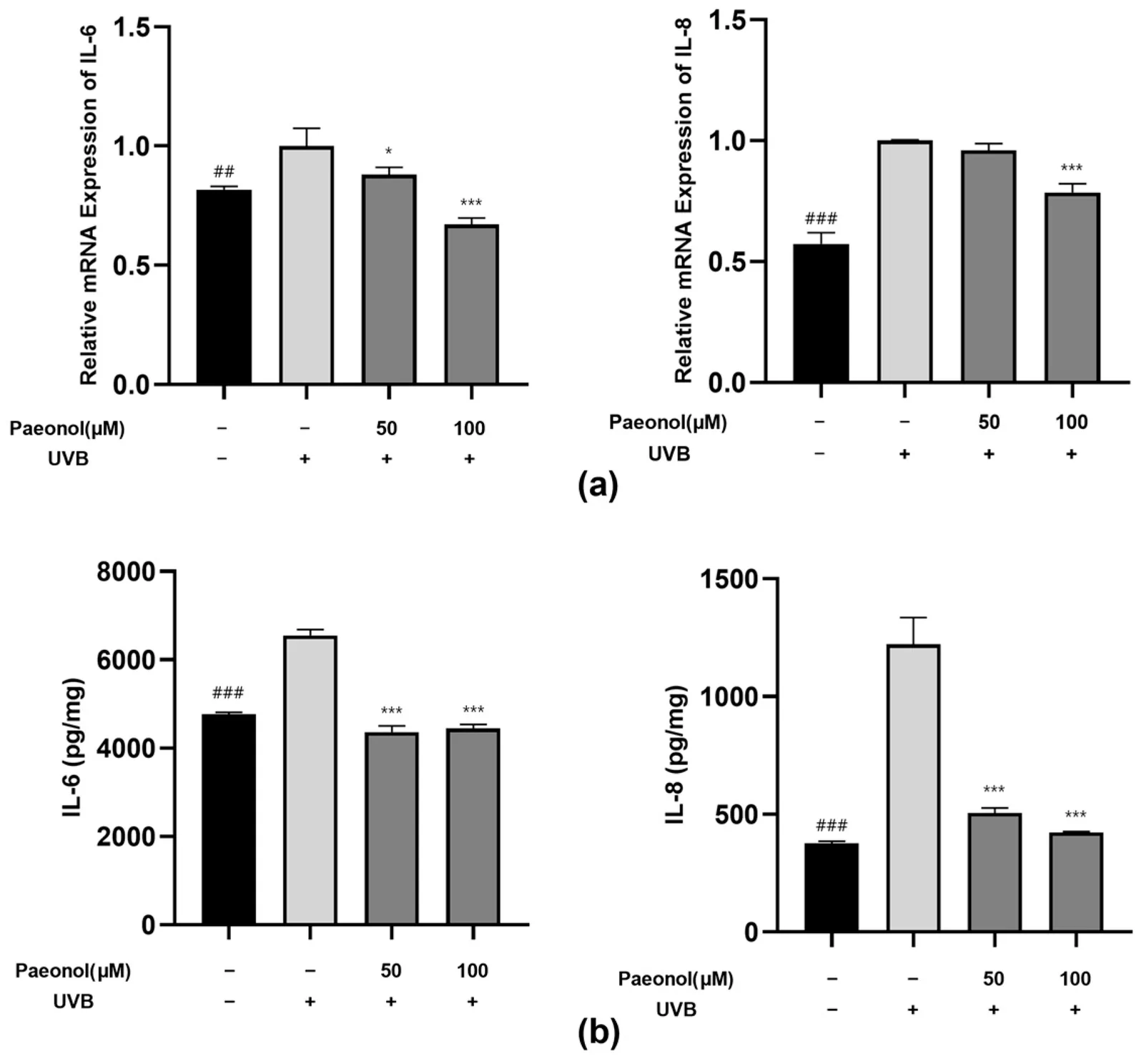
Paeonol attenuates the UVB-induced senescence-associated secretory phenotype (SASP) in HDFn cells. Senescence was induced by repeated UVB irradiation (250 mJ/cm^2^, once daily for 4 consecutive days). Candidate SASP factors were first identified by cytokine antibody array (Supplementary Figure S1) and validated for IL-6 and IL-8. (**a**) Relative mRNA expression of IL-6 and IL-8 measured by RT-qPCR; β-actin was used as the internal reference gene. (**b**) Secreted IL-6 and IL-8 protein levels quantified by ELISA. Cells were divided into the normal control, UVB control, and paeonol treatment groups (50 and 100 µM). Data are presented as the mean ± SD (n = 3). ## *p* < 0.01, ### *p* < 0.001 vs. the normal control group; * *p* < 0.05, *** *p* < 0.001 vs. the UVB control group.

Senescent fibroblasts promote skin aging by secreting a senescence-associated secretory phenotype (SASP) that drives chronic inflammation and ECM remodeling. To identify candidate SASP factors modulated by paeonol, culture supernatants were first screened using a human cytokine antibody array. UVB irradiation increased the secretion of several inflammatory mediators, most notably the CXC chemokines Gro and Gro-α, along with IL-6 and IL-8, and paeonol treatment reduced their levels (Supplementary Figure S1). Because this array was performed once (n = 1) for screening purposes, IL-6 and IL-8, two representative and functionally important SASP cytokines, were selected for quantitative validation. Consistent with the array results, paeonol significantly reduced UVB-induced IL-6 and IL-8 expression at the transcript level (Figure 4a) and at the secreted protein level (Figure 4b) in a dose-dependent manner. These findings indicate that paeonol attenuates the pro-inflammatory secretome of UVB-induced senescent fibroblasts.

### 2.5. Paeonol Attenuates UVB-Induced JNK/AP-1 Phosphorylation

**Figure 5:**
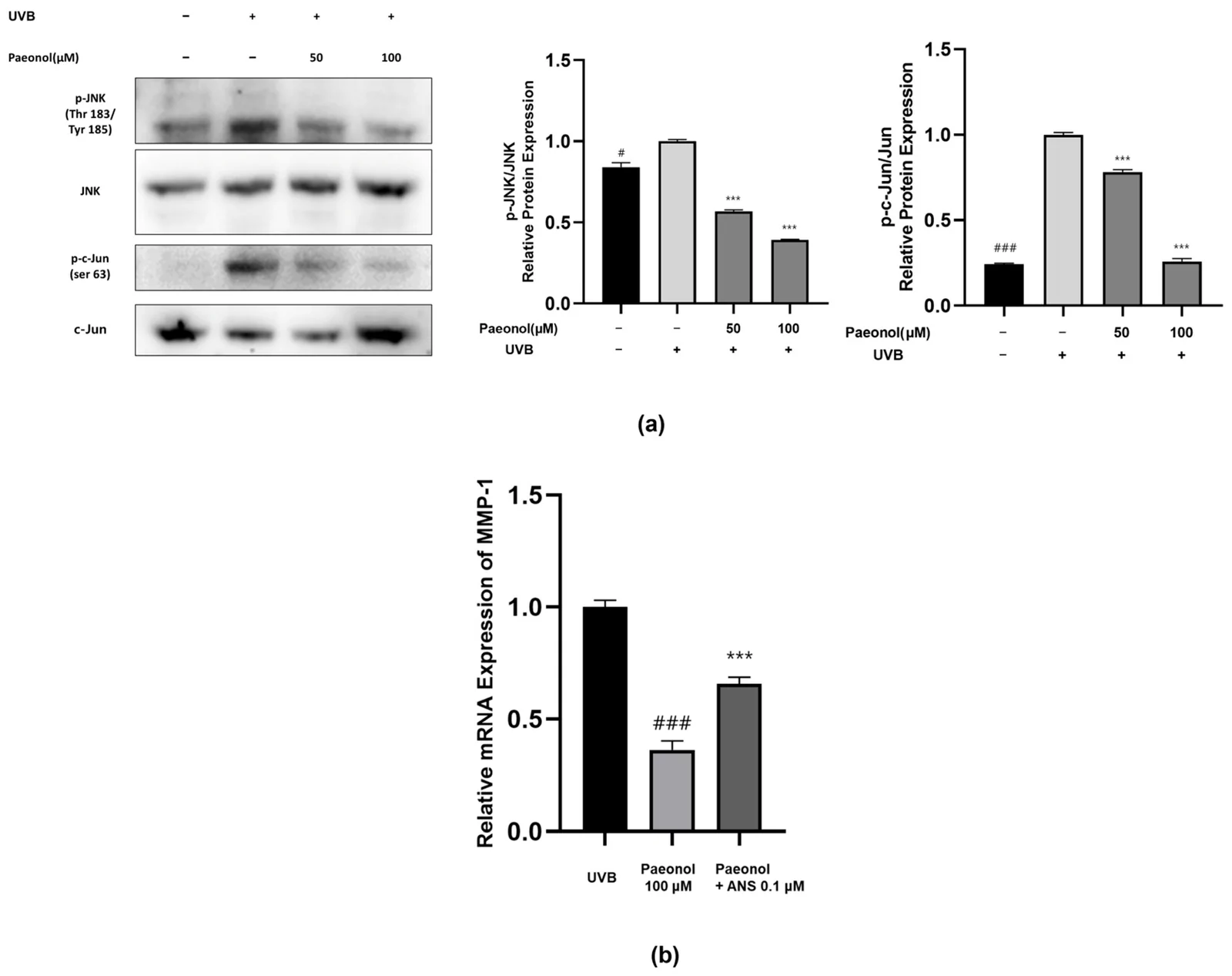
Paeonol suppresses UVB-induced JNK/AP-1 signaling in HDFn cells. Senescence was induced by repeated UVB irradiation (250 mJ/cm^2^, once daily for 4 consecutive days). (**a**) Representative Western blots of phosphorylated and total JNK and c-Jun (with β-actin as the loading control) and quantification of the p-JNK/JNK and p-c-Jun/c-Jun ratios are shown. The phospho-specific anti-bodies detected JNK (Thr183/Tyr185) and c-Jun (Ser63). UVB increased JNK and c-Jun phosphorylation, which was reduced by paeonol in a dose-dependent manner. The cells were divided into the normal control, UVB control, and paeonol treatment groups (50 and 100 µM). Data were normalized to the UVB control group. Data are presented as the mean ± SD (n = 3). # *p* < 0.05, ### *p* < 0.001 vs. the normal control group; *** *p* < 0.001 vs. the UVB control group. (**b**) Relative MMP-1 mRNA expression in cells treated with paeonol (100 µM) with or without the anisomycin (0.1 μM), measured by RT-qPCR. Data were normalized to the UVB group. Data are presented as the mean ± SD (n = 3). ### *p* < 0.001 vs. the UVB group; *** *p* < 0.001 vs. the paeonol group.

The JNK–c-Jun/AP-1 axis is a major stress-activated signaling pathway involved in regulation of MMP-1 expression and dermal matrix remodeling in dermal fibroblasts. To examine the signaling changes associated with the protective effects of paeonol, we assessed the phosphorylation of JNK and c-Jun. UVB irradiation increased the phosphorylation levels of both JNK and c-Jun relative to the normal control group, whereas paeonol treatment reduced these phosphorylation levels in a dose-dependent manner, with total JNK and c-Jun remaining unchanged (Figure 5a). The concurrent reduction in JNK/c-Jun phosphorylation and in MMP-1 expression, ROS, and SASP factors suggests that suppression of JNK/AP-1 signaling is associated with the anti-senescence and ECM-protective effects of paeonol.

To further examine the involvement of JNK signaling, cells treated with 100 μM paeonol were co-treated with the anisomycin. Anisomycin partially reversed the paeonol-mediated suppression of MMP-1 mRNA expression (Figure 5b), suggesting that the inhibitory effect of paeonol on MMP-1 is at least partly associated with suppression of JNK signaling.

## 3. Discussion

In this study, we investigated the protective effects of paeonol against UVB-SIPS in human dermal fibroblasts (HDFn) using a repeated UVB irradiation SIPS model [1,27,28,30]. Senescence was assessed using a multiparametric assessment, including SA-β-gal activity, p16, p21, p53, SASP factors, and senescent morphology, consistent with consensus recommendations for identifying cellular senescence [14,44]. PARP cleavage was examined to assess apoptosis as a potential alternative cell fate [14,15,29,30].

We assessed well-established indicators of cellular senescence. As shown by SA-β-gal staining, UVB irradiation considerably increased the fraction of senescent cells, which was reduced by paeonol treatment. This reduction was accompanied by decreased expression of key cell cycle regulators: the UVB-elevated p21WAF1 and p16INK4a were attenuated by paeonol at both the mRNA and protein levels, while p53 mRNA expression was also reduced. As p53 protein was not directly assessed, p53 activation was indirectly reflected via its transcriptional target p21 and mRNA expression, while direct p53 protein dynamics remain to be further confirmed. Paeonol also markedly reduced UVB-induced intracellular ROS accumulation, suggesting that its protective effects were accompanied by attenuation of oxidative stress. Together, these findings indicate that paeonol attenuates multiple features of the UVB-induced senescence phenotype.

Senescent fibroblasts can accelerate skin aging by secreting a senescence-associated secretory phenotype (SASP) that drives chronic inflammation and extracellular matrix (ECM) degradation [45]. An initial cytokine array screening indicated a UVB-induced SASP-associated shift, including elevation of CXC chemokines such as Gro-α/β/γ (CXCL1/2/3) and Gro-α (CXCL1), as well as IL-6 and IL-8 [27,32]. Among these candidates, IL-6 and IL-8 were validated at both the transcript and protein levels, where paeonol produced dose-dependent reductions. This anti-inflammatory profile suggests that paeonol attenuates the harmful downstream secretome of senescent cells.

UVB irradiation activates multiple mitogen-activated protein kinase (MAPK) cascades, including ERK, p38, and JNK, which can contribute to AP-1 dependent transcriptional responses [16,21]. In the present study, we focused on the JNK/AP-1 axis because JNK is an important stress responsive kinase involved in UVB-induced cellular responses, and its downstream effector c-Jun is a core component of the AP-1 complex [22,46]. Moreover, JNK/AP-1 signaling has been implicated in MMP-1 expression and dermal matrix remodeling in fibroblasts [16,33,34], making this pathway particularly relevant to our investigation of MMP-1 regulation. Consistent with this rationale, UVB irradiation increased JNK and c-Jun phosphorylation, whereas paeonol dose-dependently attenuated these changes. To further examine the functional involvement of JNK signaling, we performed a rescue experiment using anisomycin. Anisomycin partially reversed the paeonol-mediated suppression of MMP-1 mRNA expression, providing additional functional evidence that JNK signaling contributes to the regulation of MMP-1 by paeonol. Although these findings support a functional role of JNK signaling in MMP-1 regulation, they do not establish JNK/AP-1 as the sole mediator of paeonol’s protective effects, because anisomycin may affect signaling pathways beyond JNK. Although we also observed reductions in SASP factors following paeonol treatment, the extent to which these effects are mediated by JNK/AP-1 signaling remains to be established. ERK and p38 MAPK may also contribute to UVB-induced cellular responses, but they were not investigated in the present study.

Although paeonol did not enhance Type I procollagen synthesis in senescent fibroblasts, it markedly suppressed MMP-1 secretion. This indicates that paeonol preserves the dermal extracellular matrix primarily by inhibiting collagen degradation rather than by promoting its synthesis. Because senescence-associated ECM loss arises from both diminished synthesis and increased MMP-mediated breakdown [33], targeting the degradative arm represents a mechanistically coherent and physiologically meaningful protective strategy.

In conclusion, paeonol attenuated multiple features of UVB-SIPS in HDFn cells, including senescence-associated markers, inflammatory mediators, and MMP-1 production. These effects were accompanied by reduced phosphorylation of JNK and c-Jun. Furthermore, the partial reversal of paeonol-mediated MMP-1 mRNA suppression by the JNK activator anisomycin provides additional functional evidence that JNK signaling is involved in the regulation of MMP-1 expression in this model. However, these findings do not establish JNK/AP-1 signaling as the sole mediator of paeonol’s protective effects, and further investigation is needed to clarify its contribution to the broader anti-senescence effects of paeonol.

The translational relevance of these findings remains to be established because the present study employed an in vitro UVB-SIPS model that does not fully recapitulate physiological responses to solar UV radiation in vivo. Although repeated UVB irradiation is a well-established experimental approach for inducing premature senescence in dermal fibroblasts [1,27,28], UVA penetrates more deeply into the dermis and contributes substantially to chronic dermal photoaging [25,47]. Furthermore, UV radiation affects a multicellular cutaneous network and modulates local and systemic neuro–immune–endocrine pathways through signaling molecules, including hormones, neuropeptides, and cytokines, that participate in the regulation of physiological homeostasis [19,20,26]. These complex responses cannot be fully recapitulated in a fibroblast monoculture model. Therefore, further studies using UVA-based models and in vivo systems are warranted to validate the physiological and translational relevance of paeonol’s protective effects.

## 4. Materials and Methods

### 4.1. Reagents

Paeonol was purchased from Sigma-Aldrich (St. Louis, MO, USA). It was dissolved in DMSO (Biosesang, Seongnam, Republic of Korea) to prepare a 500 mM stock solution. For cell treatment, the stock solution was first diluted 1:1000 in complete culture medium and then further diluted with the medium to obtain the final working concentrations.

### 4.2. Cell Culture

Human dermal fibroblasts (HDFn) were purchased from ATCC (Manassas, VA, USA). Cells were cultured in Dulbecco’s Modified Eagle Medium (DMEM; Welgene, Gyeongsan, Republic of Korea) supplemented with 10% fetal bovine serum (FBS; Welgene, Gyeongsan, Republic of Korea) and 1% penicillin/streptomycin (Welgene, Gyeongsan, Republic of Korea) at 37 °C in a humidified atmosphere containing 5% CO_2_. All experiments were performed using cells between passages 9 and 11.

### 4.3. Cell Viability Assay

Cell viability was measured using the EZ-Cytox assay kit (DoGenBio, Seoul, Republic of Korea). HDFn were seeded in 96-well plates at a 0.8 × 10^4^ per well and treated with samples for 24 h. Then, the EZ-Cytox reagent was added to each well according to the manufacturer’s instructions. After incubation for 1 h at 37 °C in a 5% CO_2_ incubator, the absorbance was measured at 450 nm using a microplate reader (Tecan, Mannedorf, Switzerland).

### 4.4. UVB-SIPS

Stress-induced premature senescence (SIPS) was induced by repeated UVB irradiation according to a previously established method by Debacq-Chainiaux et al. [30] with modifications. Briefly, the culture medium was removed, and the cells were washed twice with Dulbecco’s phosphate-buffered saline (DPBS;Welgene, Gyeongsan, Republic of Korea). Then, the cells were covered with a thick layer of DPBS and exposed to UVB (250 mJ/cm^2^) once daily for 4 consecutive days. Irradiation was proceeded with UVB fluorescent lamps from Philips and the delivered UVB dose was confirmed using a UV dosimeter. Immediately after irradiation, DPBS was removed and replaced with fresh culture medium. After final exposure, cells were incubated for 48 h to stabilize the senescent phenotype before further experiments.

### 4.5. Experimental Design and Treatment Groups

Cells were divided into four groups: (1) normal control (no UVB, no treatment), (2) UVB control (UVB only), (3) UVB + Paeonol 50 µM, and (4) UVB + Paeonol 100 µM. For treatment groups, the cells were treated with paeonol immediately after each UVB irradiation. Then, 48 h after final UVB exposure, cells were replated. The cells were cultured for an additional 24 h before samples were collected for further experiments. The rescue experiment was performed using anisomycin (Cat. No. A9789, Sigma-Aldrich, St. Louis, MO, USA). Anisomycin was added with paeonol after replating for 48 h.

### 4.6. SA-β-Gal Staining

Senescence-associated β-galactosidase (SA-β-gal) activity was assessed using the Senescence β-Galactosidase Staining Kit (Cat. No. 9860, Cell Signaling Technology, Danvers, MA, USA) according to the manufacturer’s protocol.

### 4.7. ROS Measurement

Intracellular reactive oxygen species (ROS) levels were measured using a DCFDA/H2DCFDA Cellular ROS Assay Kit (Cat. No. ab113851, Abcam, Cambridge, UK) according to the manufacturer’s instructions. Fluorescence intensity was measured at excitation and emission wavelengths of 485 and 535 nm using a microplate reader.

### 4.8. Enzyme-Linked Immunosorbent Assay (ELISA)

After 24 h of replating, cell culture supernatants were collected and centrifuged at 655× *g* for 5 min to remove cell debris. The levels of secreted MMP-1, Pro-Collagen I alpha 1, IL-6, and IL-8 were measured using DuoSet ELISA kits (MMP-1, DY901B; Pro-Collagen I alpha 1, DY6220; IL-6, DY206; IL-8, DY208, R&D Systems, Minneapolis, MN, USA) according to the manufacturer’s instructions. ELISA data were analyzed using MyAssays online data analysis tool with a four-parameter logistic (4PL; http://www.myassays.com/four-parameter-logistic-curve.assay, accessed on 22 September 2026) model and normalized to the total protein content of each sample.

### 4.9. Reverse Transcription-Quantitative PCR (RT-qPCR)

Total RNA was extracted using RNAiso Plus (Takara Bio, Inc., Tokyo, Japan). Extracted RNA was reverse transcribed into complementary DNA (cDNA) using RevertAid Reverse Transcriptase (Thermo Fisher Scientific, Waltham, MA, USA). RT-qPCR was performed using TOPreal SYBR Green qPCR High-ROX PreMIX (Enzynomics, Daejeon, Republic of Korea) on a CFX Connect Real-Time PCR Detection System (Bio-Rad, Hercules, CA, USA). The primer sequences used in this study are listed in Supplementary Table S1. The relative gene expression was calculated using the 2^−^^ΔΔCt^ method [48], with β-actin as the internal reference gene.

### 4.10. Western Blot Analysis

Total protein was extracted using RIPA buffer (iNtRON Biotechnology, Seongnam, Republic of Korea) supplemented with PMSF (Sigma-Aldrich, St. Louis, MO, USA) and protease inhibitor cocktail (Sigma-Aldrich, St. Louis, MO, USA). The cell lysates were centrifuged at 16,343× *g* for 20 min at 4 °C, and the supernatants were collected. Protein concentration was determined using the BCA protein assay kit. Equal amounts of proteins were separated by SDS-PAGE and transferred onto PVDF membranes. The membranes were blocked with 5% bovine serum albumin (BSA; Sigma-Aldrich, St. Louis, MO, USA) in TBST for 1 h at room temperature, then incubated overnight at 4 °C with primary antibodies against p16 (#18769), p21 (#2947S), phospho-JNK (Thr183/Tyr185) (#9251S), JNK (#9252), phospho-c-Jun (Ser63) (#9261S), c-Jun (#9165S) (all from Cell Signaling Technology, Danvers, MA, USA), and β-actin was used as the loading control. After washing, the membranes were incubated with HRP-conjugated anti-rabbit or anti-mouse secondary antibodies for 2 h at 4 °C. Signals were detected using an ECL detection kit (Thermo Fisher Scientific, Waltham, MA, USA) and visualized with an iBright 1500 imaging system (Thermo Fisher Scientific). Band intensities were quantified using ImageJ software (version 1.54d, NIH, Bethesda, MD, USA).

### 4.11. Cytokine Array

Cytokine profiling was performed using a Human Cytokine Antibody Array C5 (AAH-CYT-5, RayBiotech, Peachtree Corners, GA, USA) according to the manufacturer’s instructions. Conditioned media from the normal control, UVB control, and UVB + Paeonol 100 µM groups were used. Signal intensities were quantified using excel-based analysis software provided by RayBiotech (https://www.raybiotech.com/human-cytokine-array-c5-aah-cyt-5, accessed on 22 September 2026). The data represent a single experiment conducted for screening purposes.

### 4.12. Statistical Analysis

All data are expressed as mean ± SD. Statistical analyses were performed using GraphPad Prism (version 8.0, GraphPad Software, San Diego, CA, USA). Differences between two groups (Figure 1b) were analyzed using an unpaired two-tailed Student’s *t*-test. Comparisons among multiple groups were performed using one-way ANOVA followed by Dunnett’s multiple comparison test; a *p*-value < 0.05 was considered statistically significant.

## Data Availability

No new data were created or analyzed in this study. Data sharing is not applicable to this article.

## Supplementary Materials

The following supporting information can be downloaded at https://www.mdpi.com/article/10.3390/ijms27209352/s1.

## Abbreviations

The following abbreviations are used in this manuscript:

SIPS	Stress-Induced Premature Senescence
SASP	Senescence-Associated Secretory Phenotype
