# Supplementary material for: Paeonol Attenuates UVB-Induced Premature Senescence in Human Dermal Fibroblasts to Promote Skin Longevity
Source: Int J Mol Sci. 2026 Oct 21;27(20):9352. doi: 10.3390/ijms27209352 (PMC13704950; doi:10.3390/ijms27209352)
Supplement: Supplementary file 1 [file ijms-27-09352-s001.zip › ijms-4446154-supplementary.pdf]

Paeonol Attenuates UVB-Induced Premature Senescence in Human Dermal Fibroblasts to Promote Skin Longevity

Kyuri Lee [1,†], Jeung hi Han [2,†] Hairu Zhao [1], Young Eun Kim [2], Yang Gyu Choi [2], Hosam Ki [2], Jaehong Kim [2], Young Jin Choi [3], Hye Yoon Park [3], Areum Jang [3], Min Hyuk Park [3], Hang Eui Cho [3,*] and Jaesung Hwang [1,*]

1 Department of Genetics & Biotechnology, Graduate School of biotechnology, College of Life Sciences, Kyung Hee University, Yongin 17104, Republic of Korea; kyuri.lee@khu.ac.kr (K.-R.L.); zhao@khu.ac.kr (H.-R.Z.)
2 Research and Innovation Center, LABIO Co., Ltd., Seoul 08501, Republic of Korea; msri05@labio.kr (J.-H.H.); msri14@labio.kr (Y.E.K.); msri22@labio.kr (Y.G.C.); msri07@labio.kr (H.-S.K.); msri06@labio.kr (J.-H.K.)
3 Cosmecca Korea Co., Ltd., Seongnam 13488, Republic of Korea; cyj0120@cosmecca.com (Y.J.C.); rejoice077@cosmecca.com (H.Y.P.); areumnamoo@cosmecca.com (A.-R.J.); alsgur0728@cosmecca.com (M.H.P.)
* Correspondence: bioche1975@cosmecca.com (H.E.C.); jshwang@khu.ac.kr (J.-S.H.)
† These authors contributed equally to this work.

Supplementary Materials

Supplementary Table S1. Primer sequences used for RT-qPCR

Genes	Forward Primer Sequence (5′-3′)	Reverse Primer Sequence (5′-3′)
p16	CTCGTGCTGATGCTACTG AGGA	GGTCGGCGCAGTTGGGCT CC
p21	AGGTGGACCTGGAGACT CTCAG	TCCTCTTGGAGAAGATCA GCCG
p53	CCTCAGCATCTTATCCGA GTGG	TGGATGGTGGTACAGTCA GAGC
IL-6	CCTTCTCCACAAGCGCCT TC	GGAAGGCAGCAGGCAAC A
IL-8	CCGGAAGGAACCATTCTC ACT	AGCAGGAAGGCTGCCAA GAG
β-actin	CACCATTGGCAATGAGCG GTTC	AGGTCTTTGCGGATGTCC ACGT

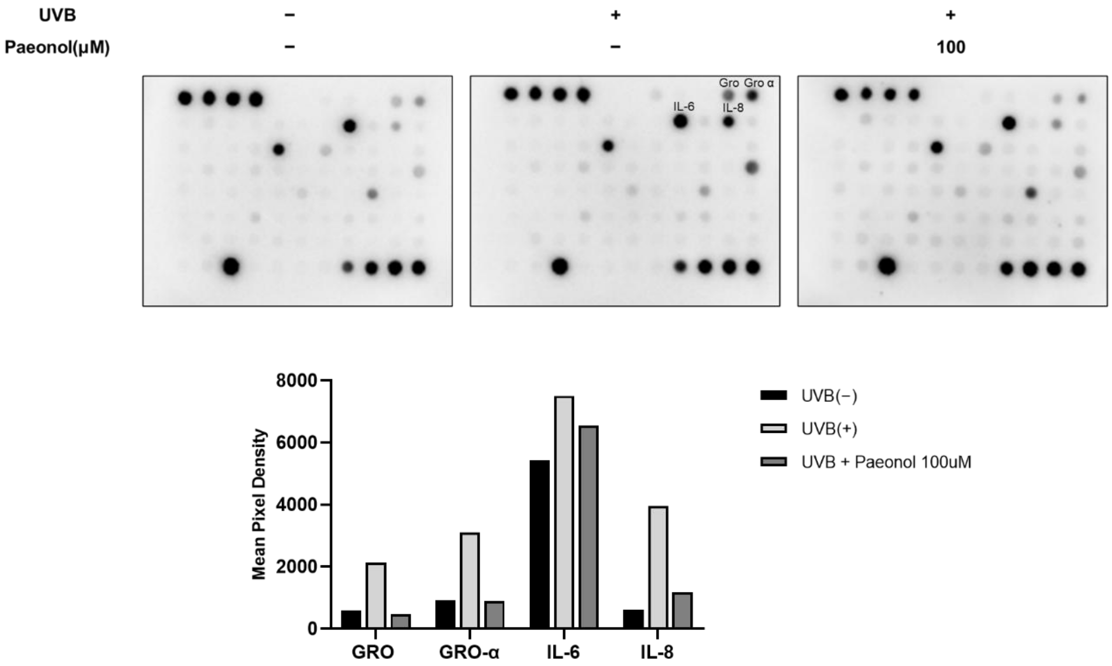

Supplementary Figure S1. SASP profiling of UVB-induced senescent HDFn cells by human cytokine antibody array. Conditioned media from the Normal control, UVB control, and UVB + paeonol (100 µM) groups were analyzed using a Human Cytokine Antibody Array C5 (AAH-CYT-5, RayBiotech). The array was performed once (n = 1) as a screening to identify candidate SASP factors. Representative array membranes and the mean pixel density of GRO, GRO-α, IL-6, and IL-8 are shown. UVB irradiation increased the levels of these factors relative to the Normal control, and paeonol treatment reduced them.
